# Effects of Free and Liposomal Doxorubicin Combined with Inductive Moderate Hyperthermia on Multimodal Intratumoural Heterogeneity in Sarcoma-45

**DOI:** 10.3390/cancers18132145

**Published:** 2026-07-03

**Authors:** Valerii B. Orel, Anatolii G. Diedkov, Valerii E. Orel, Alexandr I. Tovstolytkin, Olga Yo. Dasyukevich, Larysa M. Kovalevska, Alexander Yu. Galkin, Oleksandr Yu. Rykhalskyi

**Affiliations:** 1 National Cancer Institute, 33/43 Zdanovska Str., 03022 Kyiv, Ukraine; 2 V.G. Baryakhtar Institute of Magnetism of the National Academy of Sciences of Ukraine, 36-b Akad. Vernadskogo Blvd., 03142 Kyiv, Ukraine; 3 National Technical University of Ukraine “Igor Sikorsky Kyiv Polytechnic Institute”, 16/2 Yangel Str., 03056 Kyiv, Ukraine; 4 R.E. Kavetsky Institute of Experimental Pathology, Oncology and Radiobiology, 03022 Kyiv, Ukraine

**Keywords:** sarcoma, intratumoural heterogeneity, free doxorubicin, liposomal doxorubicin, inductive moderate hyperthermia, magnetic resonance imaging, histological examination, immunohistochemical examination, texture image analysis

## Abstract

**Background/Objectives:** Sarcomas exhibit marked intratumoural heterogeneity at the molecular, cellular and tissue levels, thereby limiting drug delivery and treatment response. Herein, we evaluated the effects of free doxorubicin (FDOX) and liposomal doxorubicin (LDOX) combined with inductive moderate hyperthermia (IMH) on intratumoural heterogeneity in an experimental sarcoma model using magnetic resonance imaging (MRI), histology and immunohistochemistry image analysis. **Methods:** Female non-inbred rats bearing sarcoma-45 were divided into six groups: control (no treatment), IMH, FDOX, LDOX, FDOX + IMH and LDOX + IMH. FDOX or LDOX was administered every other day for a total of five times, beginning from day 2 after inoculation. IMH was applied locally following drug administration using a 42 MHz radiofrequency electromagnetic field. Treatment-induced changes were assessed by T_1_- and T_2_-weighted MRI, haematoxylin–eosin–orange (H&E) staining, and Ki-67 and p53 immunohistochemistry. Moran’s spatial autocorrelation index was used as a measure of tumour phenotypic heterogeneity in medical images. **Results:** Combination treatment with different doxorubicin formulations resulted in distinct patterns of intratumoural heterogeneity in MRI and in H&E-, Ki-67- and p53-stained images. FDOX or LDOX combined with IMH produced greater deviations from the control group in multimodal spatial organisation of sarcoma-45 than the corresponding drug treatments alone. LDOX + IMH reduced tumour growth by 34% relative to the control group and was associated with distinct histological and immunohistochemical remodelling, including connective tissue replacement and the lowest Ki-67 staining level. The lowest p53 staining levels were observed in the LDOX and LDOX + IMH groups. **Conclusions:** FDOX and LDOX combined with IMH induced distinct tumour remodelling patterns in sarcoma-45. Multimodal analysis of intratumoural heterogeneity in sarcomas may provide additional quantitative information for assessing treatment response.

## Simple Summary

Sarcomas are heterogeneous tumours, with different regions of the same tumour showing variable structure at the macro- and microscopic scales. This study investigated whether combining doxorubicin, a commonly used antitumour drug, with moderate local heating produced by a non-ionising radiofrequency electromagnetic field could influence the internal spatial organisation of sarcoma. In doing so, we compared the effects of free and liposomal doxorubicin formulations combined with inductive moderate hyperthermia in rats with sarcoma-45. Tumours were assessed using magnetic resonance imaging, histology and immunohistochemistry image analyses to examine changes at tissue, cellular and molecular levels. The two drug formulations combined with inductive moderate hyperthermia produced distinct patterns of tumour remodelling. These results may contribute to quantitative approaches to evaluating treatment response in sarcoma.

## 1. Introduction

Sarcomas comprise a heterogeneous group of malignant tumours of mesenchymal origin with various histological subtypes, accounting for 1–2% of all cancers in adults and 6% in children [1]. Their rarity and diversity hinder the translation of basic research into clinical applications, thereby limiting improvements in sarcoma diagnosis and treatment. Although surgery, chemotherapy and radiotherapy remain the main treatment modalities, metastases develop in ~20–40% of patients with soft-tissue and bone sarcomas [2]. In advanced disease, chemotherapy is often the principal treatment option. However, therapeutic efficacy is limited by the marked spatial and temporal heterogeneity of sarcomas, which occurs across multiple interconnected levels of tumour organisation [3]. At the molecular level, sarcoma heterogeneity is evident in varied genetic, epigenetic, transcriptomic and proteomic profiles [4]. At the cellular level, it is associated with phenotypically distinct malignant cell subpopulations that exhibit variable mitochondrial metabolism, oxidative stress responses and susceptibility to cell death [5]. At the tissue level, heterogeneity manifests itself as the spatial distribution of tumour cells and their interactions with components of the tumour microenvironment [6]. More broadly, intratumoural heterogeneity across macro- and microscales is shaped by interactions of physical, chemical and biological phenomena.

Much current sarcoma research is directed at understanding the complex interactions of biophysical, biochemical and pathophysiological processes in the tumour and the surrounding tissues, which give rise to heterogeneity across molecular, cellular, tissue and whole-tumour scales. Biophysical processes encompass the effects of mechanical forces, elasticity and tissue stiffness, as well as perturbations linked to mitosis and replication stress [7]. Biochemical processes mediated by oxidative stress lead to chromosomal rearrangements, base modifications, DNA strand breaks and crosslinking [8]. Pathophysiological processes involve dysregulation of cell signalling pathways, variation in gene expression, epigenetic modification and the remodelling of the tumour microenvironment [9]. Collectively, these multilevel processes influence not only sarcoma development and heterogeneity but also its response to treatment, under the selective pressure of which resistant subpopulations may emerge through alterations in drug targets or activation of compensatory pathways. The relative abundance of tumour cell clones with different mechanisms of chemotherapy and radiotherapy resistance can change over time, contributing to both spatial and temporal heterogeneity [10]. Moreover, regional variation in tissue architecture, cellular composition, vascularisation, interstitial pressure and microenvironment impairs drug penetration and produces spatially non-uniform treatment effects [11].

Free doxorubicin (FDOX), the conventional non-liposomal formulation, is widely used as a chemotherapeutic agent in multimodal management of high-grade osteosarcomas and advanced soft-tissue sarcomas, while its clinical efficacy is constrained by limited tumour selectivity and systemic toxicity. For this reason, liposomal nanoformulation of doxorubicin (LDOX) has been designed to reduce toxic side effects and improve drug delivery to heterogeneous solid tumours. Assessment of therapeutic efficacy should incorporate imaging approaches that evaluate intratumoural heterogeneity. Medical imaging modalities, including X-ray, ultrasound, computed tomography, positron emission tomography and magnetic resonance imaging (MRI), enable quantitative characterisation of treatment-specific changes in intratumoural heterogeneity [12]. Quantitative image analysis can capture biologically relevant tumour phenotypes and spatial heterogeneity across multiple scales, providing imaging-derived surrogates of tissue architecture, cellular composition and microenvironmental organisation. This concept forms the basis of radiomics and radiogenomics, which seek to relate imaging features to tumour biology and treatment response [13,14]. Such differences between FDOX and LDOX are expected to reflect variations in pharmacokinetics, intratumoural drug distribution, cellular damage and death, as well as the remodelling of the tumour microenvironment and drug resistance [15].

Combination treatment strategies have therefore been proposed to target multiple sources of therapeutic resistance. One such modality is inductive moderate hyperthermia (IMH), which is commonly combined with chemotherapy to enhance the antitumour effect on the primary lesion or a solitary metastasis [16,17]. The rationale for combining FDOX or LDOX with IMH is based on thermal (local heating at <42 °C increases blood flow, vascular permeability and drug release, altering cellular membrane properties) as well as nonthermal (electromagnetic fields influence ionic transport and free radical reaction rates, enhancing oxidative stress) effects of non-ionising electromagnetic irradiation [18,19,20]. Yet the effects of combination treatment depend critically on the thermal dose, drug formulation, scheduling and spatial distribution of electromagnetic fields within the tumour region. Careful characterisation of these factors is thus required to minimise side effects [21]. Multiscale visualisation enables quantitative analysis of intratumoural heterogeneity across various levels. By combining imaging modalities and integrating their interrelated datasets from the molecular to the tissue level, a more comprehensive understanding of therapeutic response can be achieved [22]. This is particularly relevant to sarcomas, in which heterogeneous drug delivery, microenvironment variation and treatment-induced changes have a substantial impact on patient outcomes.

The present work builds on our previous studies, in which we compared the effects of FDOX and LDOX in combination with IMH in vitro [23,24] and investigated the effects of IMH alone on intratumoural heterogeneity of sarcoma in vivo [25]. Given the importance of sarcoma heterogeneity in diagnosis, treatment and prognosis, the present study aims to evaluate the effects of FDOX and LDOX combined with IMH on intratumoural heterogeneity in sarcoma-bearing animals using MRI, histological and immunohistochemical image analyses.

## 2. Materials and Methods

### 2.1. Experimental Animals and Sarcoma-45 Growth Kinetics

Female non-inbred rats, maintained in the vivarium of the National Cancer Institute (NCI; Ukraine), were randomly divided into six groups (*n* = 6 in each): (1) sarcoma-bearing animals receiving no treatment (control group); (2) sarcoma-bearing animals exposed to IMH alone (IMH); (3) sarcoma-bearing animals treated with FDOX alone (FDOX); (4) sarcoma-bearing animals treated with an FDOX and IMH combination (FDOX + IMH); (5) sarcoma-bearing animals treated with LDOX alone (LDOX); (6) sarcoma-bearing animals treated with an LDOX and IMH combination (LDOX + IMH). Group sizes were selected in accordance with established guidelines for laboratory animal experiments and ethical principles aimed at minimising animal use while maintaining scientific validity [26]. Animals were housed under controlled environmental conditions (room temperature of 20–24 °C; relative humidity of 50–60% and a 12 h light/12 h dark cycle) and kept in standard cages with bedding material. Standard laboratory chow and water were available ad libitum. Housing and husbandry conditions were maintained following the ARRIVE 2.0 recommendations for animal research [27,28]. Rat sarcoma-45 cells were obtained from the tumour strain bank of the R.E. Kavetsky Institute of Experimental Pathology, Oncology and Radiobiology, NAS of Ukraine (Kyiv, Ukraine). The tumour was induced by a subcutaneous injection of 0.5 mL of sarcoma-45 cell suspension containing 1 × 10^6^ cells into the right hindlimb. This animal model comprises spindle cells with numerous mitotic figures and resembles high-grade undifferentiated pleomorphic sarcoma, a common subtype of soft-tissue sarcomas in adults [29,30,31]. Tumour-bearing rats were given either FDOX (Ebewe, Unterach, Austria) at a dose of 1.5 mg/kg or LDOX (Dr. Reddy’s Laboratories, Hyderabad, India) at a dose of 2 mg/kg body weight, so that the concentration of the active agent was equivalent in the two formulations. The selected doses were based on previously published preclinical studies. The FDOX dose was adopted from studies using the Walker-256 carcinosarcoma model, whereas the LDOX dose was selected based on evidence of altered pharmacokinetics and lower systemic toxicity in relation to the free formulation. These doses were chosen to ensure comparable therapeutic exposure to doxorubicin while maintaining acceptable tolerability [32,33]. The drugs were administered by intravenous injection every other day for a total of five times, beginning on day 2 after inoculation. The experiments were designed in accordance with the research recommendations reported in [34]. All procedures were approved by the Institutional Committee for Animal and Medical Research Ethics. Where applicable, this study was conducted in compliance with Law of Ukraine No. 3447-IV and European Directive 2010/63/EU.

Tumour volumes were calculated from calliper-derived measurements of length (L), width (W) and height (H) using the ellipsoid formula [35]:

(1) V = L × W × H × π/6,

where L is the length, W is the width and H is the height of the tumour.

To compare the effects of different treatment modalities on sarcoma-45 growth, we calculated the growth factor φ and breaking ratio κ, parameters that consider the role of free radicals in nonlinear tumour growth kinetics [36]. These parameters are appropriate because FDOX, LDOX and IMH are known to influence free radical generation and oxidative stress [37,38].

### 2.2. Inductive Moderate Hyperthermia

IMH was applied using an experimental prototype of the MagTherm medical device (Radmir, Kharkiv, Ukraine), operated at a frequency of 42 MHz and a power of 20 W. Electromagnetic exposure was localised to the tumour region in sarcoma-bearing animals by using an applicator comprising a loop and ferromagnetic dipoles (NCI, Kyiv, Ukraine). The applicator size could be adjusted to match the tumour shape and size in a personalised manner [39]. Since heating was more pronounced adjacent to the applicator than at the loop centre, this design was advantageous for targeting viable and proliferating cells, predominantly found at the tumour periphery [40,41]. IMH treatment consisted of five sessions, each lasting 15 min, delivered every other day starting on day 2 after tumour inoculation. In the FDOX + IMH and LDOX + IMH groups, the electromagnetic field was applied directly following drug administration. Before each treatment procedure, tumour-bearing animals were anaesthetised with 1–2% isoflurane and immobilised in the prone position.

The maximum intratumoural temperature during IMH was measured using TM-4 fibre-optic thermometers (Radmir, Ukraine) and an uncooled thermal camera (ThermaCAM E300, FLIR Systems, Wilsonville, OR, USA; sensitivity, 0.1 °C; spectral range, 8–14 μm). The fibre-optic probe was positioned approximately at the centre of the tumour while the animals were under anaesthesia. Temperature increased rapidly during the first 8–10 min of treatment and then reached a stable plateau of ~39 °C for the remainder of the 15 min IMH session, without pronounced local temperature maxima across the tumour surface. The temperature distribution had a standard deviation of ~1.57 °C and a Moran’s spatial autocorrelation index of 0.49 a.u., indicating moderate heterogeneity of heat distribution with retained spatial clustering. In general, these observations were consistent with the COMSOL simulations (Table 1), which also predicted temperatures < 42 °C. The slightly higher computed temperature in the skin region of interest (ROI) was expected because of its proximity to the applicator, whereas the tumour ROI exhibited the highest specific absorption rate (SAR). Although the achieved temperature was below the conventional hyperthermia threshold, previous studies indicate that radiofrequency hyperthermia can exert biological effects beyond those attributable to mild heating alone, consistent with a contribution from nonthermal mechanisms [42,43,44]. This may improve patient tolerability by reducing heat-associated pain.

**Figure 1:**
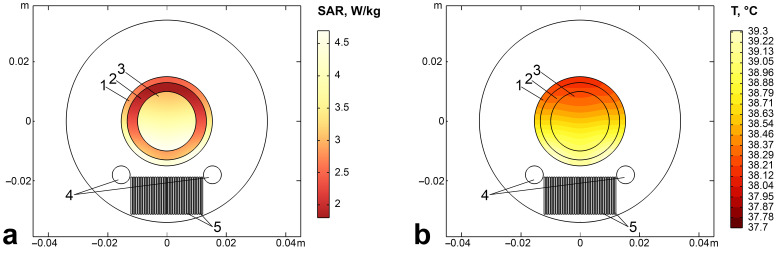
Computational modelling of specific absorption rate (**a**) and temperature distribution (**b**) in sarcoma-45 in response to IMH: 1—skin; 2—subcutaneous tissue; 3—soft-tissue tumour; 4—loop applicator; 5—magnetic dipoles.

To optimise the treatment plan, IMH was simulated in COMSOL Multiphysics v. 5.6 (COMSOL AB, Stockholm, Sweden) software by coupling the AC/DC and heat-transfer modules. Circular ROIs, encompassing the tumour, subcutaneous tissues and skin, were created based on magnetic resonance imaging (MRI) data acquired from the animals. The model was built using the finite-element method, incorporating the tissue properties of density, heat capacity, thermal and electrical conductivity, and relative permittivity taken from [45,46,47,48]. Figure 1 shows the spatial distributions of SAR and temperature in response to IMH.

### 2.3. Magnetic Resonance Imaging

MRI data were acquired using a 1.5 T scanner (Intera, Philips, Amsterdam, The Netherlands) equipped with an eight-channel knee coil array (SENSE Knee Coil, Philips). Before imaging, the rats were anaesthetised and immobilised as described above. For each animal, T_1_-weighted (T_1_W) and T_2_-weighted (T_2_W) images were obtained using the acquisition parameters listed in Table 2. MRI features characteristic of treatment response in soft-tissue sarcomas were evaluated according to [49].

### 2.4. Histological Examination

After induction of anaesthesia as described above, the animals were euthanised in accordance with the approved protocol. The tumours were excised and fixed in 10% neutral-buffered formalin for 7 days. Then, the specimens were processed for paraffin embedding, sectioned at 5 μm, mounted on glass slides, and stained with haematoxylin–eosin–orange (H&E), as described in [50]. Histological samples were imaged and assessed according to [51] using a 40–1600× trinocular infinity-corrected microscope with LED illumination (AmScope, Irvine, CA, USA), fitted with an MU1000-3PL 10 MP digital camera (AmScope, USA). A semi-quantitative analysis of resected sarcomas was carried out by a single pathologist blinded to the preliminary findings and treatment allocation. Common histopathological features were evaluated within one section per centimetre of the largest tumour diameter, following recommendations from [52,53,54]: necrosis, defined as loss of cellular and nuclear detail, ghost-cell appearance, nuclear and cellular debris; inflammation, defined as infiltration of inflammatory cells within stromal regions; apoptosis, defined as cellular shrinkage, condensed or fragmented hyperchromatic nuclei and apoptotic bodies; connective tissue replacement, defined as eosinophilic fibrotic stromal tissue, including collagen-rich fibres or bands containing spindle-shaped stromal cells; fatty tissue replacement, defined as adipose-like tissue containing clear cytoplasmic vacuoles and peripherally displaced nuclei. Briefly, to assess lesions for feature severity, histological images were assigned a score ranging from 0 (not observed or <10%) to 1 (mild < 50%), 2 (moderate < 70%) and 3 (severe > 70%) of the field of view based on [55,56,57,58].

Immunohistochemical staining for Ki-67 and p53 was performed on deparaffinized sections dried for 1 h at 56 °C according to the recommendations [59]. For antigen detection, extraction was performed using 10 mM sodium citrate (pH 4.0) heated for 30 min at 95° C in a water bath. The expression of these proteins in tissue sections was detected using specific antibodies against Ki-67 (clone SP6) rabbit monoclonal antibodies and p53 (clone PAb 240), diluted 1:200 in 2% BSA-PBS overnight in a humidified chamber. Before incubation with antibodies, endogenous peroxidase activity was blocked with hydrogen peroxide, after which the sections were incubated in normal goat serum to minimise nonspecific antibody binding. Detection was performed using the EnVision system (DAKO, Santa Clara, CA, USA) with a second 30 min incubation step. After washing in phosphate-buffered saline, peroxidase activity was visualised using diaminobenzidine (DAB). The sections were then counterstained with haematoxylin for 1–2 min and mounted in balm. Expression was assessed using H-score [60].

### 2.5. Imaging Analysis of Intratumoural Heterogeneity

MRI scans were segmented and quantitatively analysed in Fiji software (ImageJ2 v. 2.14, NIH, Bethesda, MD, USA), following [61,62]. For each MRI dataset, semi-automated segmentation was undertaken by a single radiologist, blinded to treatment allocation, using initial maximum entropy thresholding followed by manual contour correction [63]. Two ROIs were delineated: (i) the tumour, identified as a mass with an iso- to hyperintense signal on T_1_W and T_2_W images; (ii) the hamstring muscle of the contralateral limb, identified by its isointense signal on T_1_W and T_2_W images. Segmentation of deconvoluted H&E-stained images was performed using the ColorDeconvolution2 and K-means clustering plugins in Fiji software prior to image heterogeneity analyses [64,65]. For immunohistochemical images, the haematoxylin and DAB channels were first separated using the ColorDeconvolution2 plugin to assess Ki-67 and p53 staining levels based on mean image brightness and were then converted into HSB colour space using the ColorSpaceConverter plugin in Fiji software [66,67]. Moran’s spatial autocorrelation index (Moran’s I) was subsequently calculated as a quantitative measure of spatial texture heterogeneity in MRI, histological and immunohistochemical images using Autocorrelation v.1.0 software (NCI) [68,69,70]. Moran’s I reflects the spatial organisation of imaging-derived phenotypic features within the tumour. Although it does not directly measure genetic, clonal or molecular heterogeneity, previous studies have shown that spatial autocorrelation metrics contribute biologically relevant information from MRI and histological image analyses [71,72,73,74]. In the present study, we interpreted Moran’s I in relation to modality-specific mechanisms of image contrast. Higher Moran’s I values denote stronger spatial autocorrelation, corresponding to a more clustered or homogeneous image texture, whereas lower Moran’s I values indicate weaker spatial autocorrelation and a more heterogeneous image texture [75].

### 2.6. Statistical Analysis

Normality was assessed using the Shapiro–Wilk and Kolmogorov–Smirnov tests. Statistical comparisons of experimental groups were performed using one-way ANOVA followed by the Games–Howell post hoc test or by the Kruskal–Wallis H test followed by Dunn’s post hoc test with Bonferroni correction. Differences among ROIs within a single group were analysed using either Student’s two-tailed *t*-test or the Mann–Whitney U test, as appropriate. Values of *p* < 0.05 were considered statistically significant. All analyses were conducted using SPSS Statistics v. 25.0 (IBM Inc., Armonk, NY, USA; 2017) and Jamovi v. 2.6 (Jamovi project; 2025).

## 3. Results

### 3.1. Sarcoma-45 Growth Kinetics

**Figure 2:**
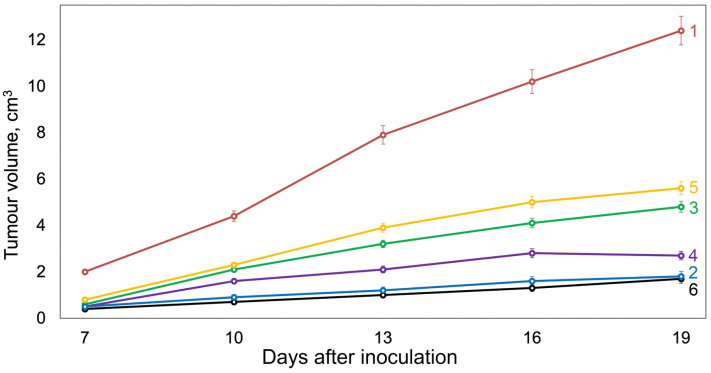
Nonlinear tumour growth kinetics of sarcoma-45: 1—control (no treatment); 2—IMH; 3—FDOX; 4—FDOX + IMH; 5—LDOX; 6—LDOX + IMH.

Figure 2 shows the tumour growth kinetic curves, and Table 3 provides the growth factors and braking ratios in sarcoma-bearing animals. Treatment with FDOX or LDOX alone inhibited tumour growth by 21% and 17%, respectively, when compared with the control group. Applying IMH with FDOX or LDOX resulted in a more pronounced antitumour effect, reducing the growth factor by 26% and 34%, respectively, relative to the control group. The lowest growth factor and the highest braking ratio were observed in the LDOX + IMH group; however, these findings should be interpreted as indicating a distinct biological response pattern rather than superior antitumour efficacy relative to IMH alone. As these findings reflect only macroscopic tumour growth, the structural and biological characteristics of the residual sarcoma-45 tissue were examined further.

### 3.2. Magnetic Resonance Imaging of Intratumoural Heterogeneity in Sarcoma-45

**Figure 3:**
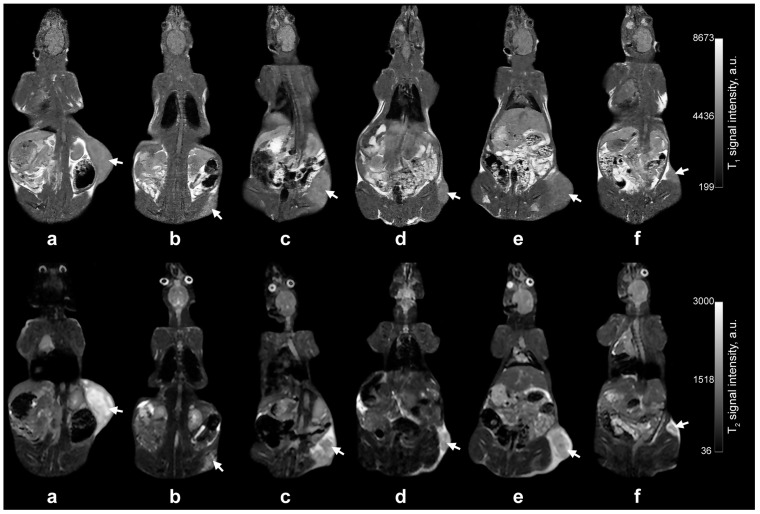
Representative coronal T_1_-weighted (**top row**) and T_2_-weighted (**bottom row**) MRI scans of sarcoma-45-bearing rats on day 19 after tumour inoculation: (**a**) control (no treatment); (**b**) IMH; (**c**) FDOX; (**d**) FDOX + IMH; (**e**) LDOX; (**f**) LDOX + IMH. White arrows indicate tumour ROIs.

Figure 3 shows representative coronal T_1_W and T_2_W images of sarcoma-45-bearing rats on day 19 after tumour inoculation. Sarcoma-45 appeared as a superficial oval mass with heterogeneous signal characteristics, demonstrating predominantly higher signal intensity than the muscle tissue in the contralateral limb.

The control group exhibited MRI features suggestive of central necrosis and extensive peritumoural oedema, which mirrors earlier findings that soft-tissue sarcomas outgrow their vascular supply, resulting in infarction, necrosis and heterogeneous T_2_W signal intensity [76]. In response to IMH treatment alone, the tumours appeared as linear band-like lesions with intermediate-to-high signal intensity on T_1_W and T_2_W images, compatible with post-treatment fibrotic changes on MRI [77]. The FDOX + IMH and LDOX + IMH groups had more defined tumour margins and less extensive peritumoural oedema than those in FDOX or LDOX. Such qualitative MRI findings have previously been associated with improved outcomes in patients with soft-tissue sarcoma following neoadjuvant treatment [49,78,79].

**Figure 4:**
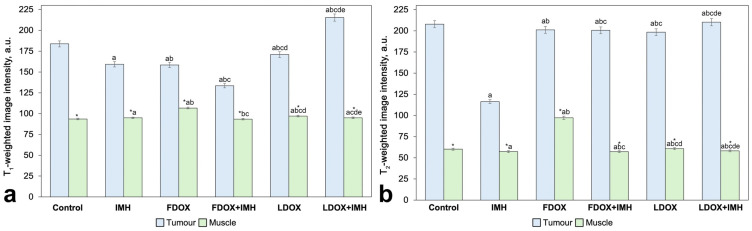
Sarcoma-45 signal intensity on T_1_-weighted (**a**) and T_2_-weighted (**b**) MRI scans. * Statistically significant difference from the tumour ROI in the corresponding group, *p* < 0.05; ^a^ statistically significant difference from the control (no treatment), *p* < 0.05; ^b^ statistically significant difference from IMH, *p* < 0.05; ^c^ statistically significant difference from FDOX, *p* < 0.05; ^d^ statistically significant difference from FDOX + IMH, *p* < 0.05; ^e^ statistically significant difference from LDOX, *p* < 0.05.

Quantitative analysis of ROI signal intensity and intratumoural heterogeneity in sarcoma-45 T_1_W and T_2_W images is summarised in Figure 4 and Table 4. Tumour ROIs and the corresponding contralateral muscle ROIs demonstrated marked differences (*p* < 0.05) in signal intensity and Moran’s I on both T_1_W and T_2_W sequences. Muscle ROIs, however, did not differ significantly in Moran’s I (0.34 ± 0.01 a.u.) between animal groups in any of the sequences (*p* > 0.05).

On T_1_W images, the tumour-to-muscle signal intensity ratio was lower, on average, by 19% in response to IMH, FDOX, FDOX + IMH and LDOX treatment, compared with the control group. Conversely, LDOX + IMH increased the tumour-to-muscle ratio by 16%. Moran’s I extracted from tumour ROIs was substantially smaller in all treated groups than in the control group. The minimum Moran’s I values were observed after IMH alone and LDOX + IMH; there were no statistically significant differences between the two groups. FDOX and LDOX alone reduced Moran’s I by 16% and 12%, respectively, whereas their combination with IMH led to greater declines, 20% for FDOX + IMH and 26% for LDOX + IMH, relative to the control group (*p* < 0.05). We did not find any significant difference in Moran’s I between FDOX and LDOX action alone.

On T_2_W images, changes in tumour ROI parameters differed from those on T_1_W. IMH action caused the lowest signal intensity and Moran’s I of the tumour ROI. FDOX and LDOX elevated tumour ROI Moran’s I by 27%, on average, relative to the control group. Combining IMH with either FDOX or LDOX reduced Moran’s I by approximately 10% compared with the respective drug formulation alone; however, Moran’s I in both combination groups remained higher than in the untreated control group (*p* < 0.05).

Together, qualitative and quantitative MRI analyses showed that IMH modified heterogeneity within the tumour ROI and produced radiological features in T_1_W and T_2_W images consistent with treatment-induced remodelling.

### 3.3. Histological and Immunohistochemical Assessment of Intratumoural Heterogeneity in Sarcoma-45

**Figure 5:**
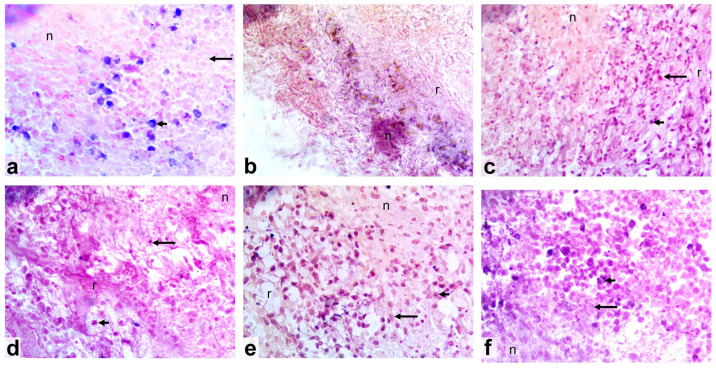
Histological findings observed in sarcoma-45 (H&E, ×400): (**a**) control (no treatment); (**b**) IMH; (**c**) FDOX; (**d**) FDOX + IMH; (**e**) LDOX; (**f**) LDOX + IMH. n—necrotic regions; r—connective tissue replacement foci; i—inflammatory infiltration; short arrows—nucleated cells; long arrows—non-nucleated cells, karyorrhexis.

Morphological features observed in sarcoma-45 are shown in Figure 5 and Table 5. In the control group (Figure 5a), the tumours comprised predominantly eosinophilic non-nucleated cells, although large cells with hypochromic nuclei were also present, indicating chromatin decondensation in tumour cells with high metabolic activity [80]. Necrotic masses accounted for ~50% of the tissue, and there were occasional areas of haemorrhage. In response to IMH (Figure 5b), the tumours appeared to have connective tissue fibres and necrotic masses, with few residual cells. After FDOX treatment (Figure 5c), there were areas of necrosis surrounded by connective tissue fibres, which were infiltrated by eosinophilic nucleated and non-nucleated cells. In the FDOX + IMH group (Figure 5d), the tumours demonstrated infiltration of the connective tissue fibres with approximately equal proportions of eosinophilic nucleated and non-nucleated cells, while hypochromic nuclei were rare. LDOX treatment (Figure 5e) resulted in extensive necrosis accounting for ~70% of the tissue, though some connective tissue fibres were also seen. Most cells were eosinophilic and non-nucleated, compatible with treatment-associated cell loss and necrotic or apoptotic changes. In the LDOX + IMH group (Figure 5f), there were extensive areas of connective tissue replacement and necrosis occupying ~50% of the tissue, involving inflammatory-cell infiltration and minor inclusions of adipose tissue (~10%). These morphological findings confirm the treatment-dependent remodelling of sarcoma-45 tissue, with varying degrees of necrosis, connective tissue replacement and residual cellularity. To further characterise the biological activity of the residual tumour component, immunohistochemical staining for Ki-67 and p53 was performed.

**Figure 6:**
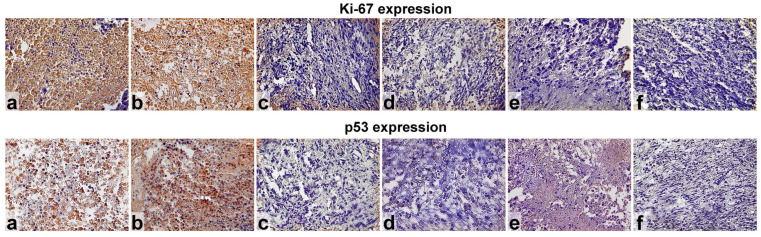
Immunohistochemical staining of Ki-67 and p53 in sarcoma-45 (×400): (**a**) control (no treatment); (**b**) IMH; (**c**) FDOX; (**d**) FDOX + IMH; (**e**) LDOX; (**f**) LDOX + IMH.

**Figure 7:**
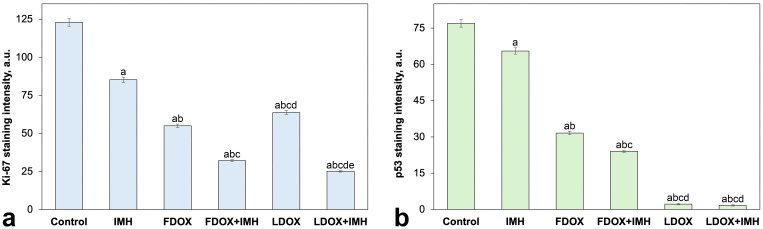
Staining intensity of Ki-67 (**a**) and p53 (**b**) in sarcoma-45. ^a^ Statistically significant difference from control (no treatment), *p* < 0.05; ^b^ statistically significant difference from IMH, *p* < 0.05; ^c^ statistically significant difference from FDOX, *p* < 0.05; ^d^ statistically significant difference from FDOX + IMH, *p* < 0.05; ^e^ statistically significant difference from LDOX, *p* < 0.05.

As shown in Figure 6 and Figure 7, the control group was characterised by the highest Ki-67 and p53 staining levels. IMH alone caused 31% and 15% decrease in Ki-67 and p53, respectively, relative to the control group. Compared with the control, FDOX and LDOX treatment reduced Ki-67 staining by 55% and 48%, respectively (*p* < 0.05). In addition, the combination of FDOX with IMH reduced both Ki-67 and p53 compared with FDOX alone. Among the treatments given, LDOX + IMH induced the minimum Ki-67 level in sarcoma-45. For p53, the lowest staining level was detected in the LDOX and LDOX + IMH groups. Combining LDOX with IMH did not initiate a further decrease in p53 levels, compared with LDOX alone.

Analysis of intratumoural heterogeneity in histological and immunohistochemical images of sarcoma-45 is presented in Table 6. In H&E-stained images, the minimum Moran’s I value was recorded following FDOX + IMH treatment, consistent with the greatest degree of tissue heterogeneity [81]. In Ki-67- and p53-stained images, the untreated control group had the lowest Moran’s I values, which reflected the most heterogeneous spatial distributions of Ki-67 and p53 proteins in sarcoma-45 cells [82,83]. IMH alone initiated a 2.1-fold and 1.3-fold rise in Moran’s I extracted from H&E- and Ki-67-stained images, respectively, relative to the control group, whereas it had no significant effect on p53-stained images. FDOX did not substantially alter spatial autocorrelation in histological images but increased Moran’s I in Ki-67- and p53-stained images with respect to the control group (*p* < 0.05). When comparing FDOX and FDOX + IMH groups, we found that the combination treatment reduced Moran’s I by 48% in H&E-stained images and by 7% in Ki-67-stained images, accompanied by a 70% increase in p53-stained images. In contrast, adding IMH to LDOX induced a 20% and 26% higher Moran’s I in H&E-stained and Ki-67-stained images, respectively. No statistically significant difference in the spatial pattern of p53 was detected between the LDOX and LDOX + IMH groups. Notably, FDOX and LDOX alone had similar effects on the spatial pattern of p53 (*p* > 0.05).

Overall, these results indicated treatment-dependent differences in sarcoma-45 histological and immunohistochemical heterogeneity. IMH alone and in combination with either FDOX or LDOX altered the distribution patterns of tissue components, as well as Ki-67 and p53 staining, while image-derived tumour heterogeneity profiles differed between the FDOX + IMH and LDOX + IMH treatment groups.

## 4. Discussion

**Figure 8:**
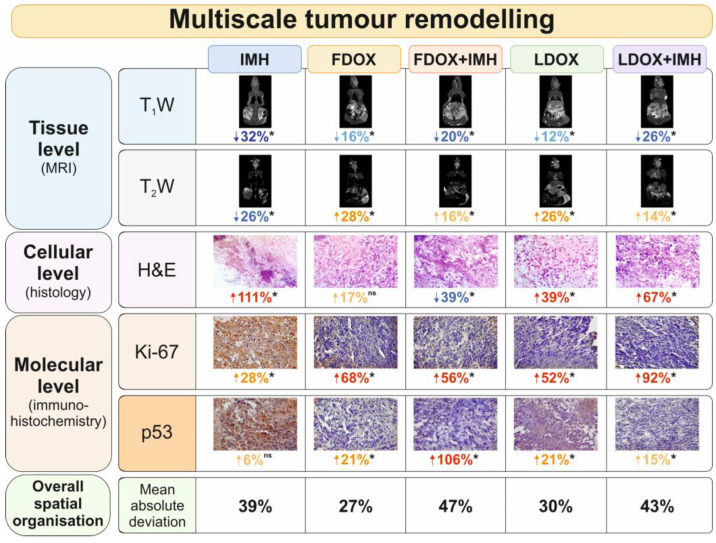
Comparative analysis of treatment-induced changes in intratumoural heterogeneity of sarcoma-45 across tissue, cellular and molecular levels of organisation. The tissue level was assessed using T_1_-weighted (T_1_W) and T_2_-weighted (T_2_W) MRI scans; the cellular level was assessed using haematoxylin–eosin–orange (H&E)-stained histological images; and the molecular level was assessed using Ki-67- and p53-stained immunohistochemical images. Percentages and arrows indicate the magnitude and direction of changes in Moran’s spatial autocorrelation index relative to the untreated control group. * Significant difference from the control (no treatment) group, *p* < 0.05; ^ns^ non-significant difference from the control (no treatment) group, *p* > 0.05. Mean absolute deviation represents the mean of the absolute percentage deviations in Moran’s index from the untreated control group, calculated for modalities with statistically significant differences, and reflects the magnitude of treatment-associated spatial reorganisation irrespective of the direction of change.

Several studies have previously compared the antitumour effects of FDOX and LDOX in combination with hyperthermia in experimental sarcoma models [84,85,86]. Intratumoural heterogeneity has also been reported to increase or decrease in response to treatment, depending on tumour biology and the imaging method employed [87,88,89]. Herein, we investigated the combined effects of FDOX and LDOX with IMH on sarcoma-45 intratumoural heterogeneity at the tissue, cellular and molecular levels of organisation. The untreated control group served as the baseline for evaluating treatment-induced changes. In doing so, we performed qualitative and quantitative analyses of MRI, histological and immunohistochemical images, which are routinely acquired during the clinical assessment of patients with sarcoma. Figure 8 summarises treatment-induced changes in spatial tumour organisation across imaging modalities. When compared with the control group, FDOX + IMH and LDOX + IMH produced mean absolute deviations of 47% and 43% in multimodal intratumoural heterogeneity, as measured by Moran’s I, respectively. In the following paragraphs, we discuss the influence of FDOX or LDOX combined with IMH on multimodal intratumoural heterogeneity in sarcoma-45.

At the tissue level visualised by MRI, treatment groups showed lower Moran’s I on T_1_W images, indicating greater heterogeneity in tumour texture. This effect was the greatest following treatment with IMH alone or LDOX + IMH. By contrast, analysis of T_2_W images showed higher Moran’s I values, consistent with more homogeneous texture, across treatment groups, except for IMH. These divergent patterns on T_1_W and T_2_W imaging reflect the sensitivity of the two sequences to different components of tissue remodelling, including changes in oedema, necrosis, granulation tissue and fibrosis [90]. Signal intensity on T_1_W and T_2_W images depends on the biophysical and biochemical environment of hydrogen protons within the tissue. T_1_ signal hyperintensity (Figure 4a) commonly arises from the release of macromolecules (e.g., proteins) and paramagnetic ions during cell and tissue necrosis, as well as from fat inclusions, all of which shorten the spin–lattice relaxation time of hydrogen protons [91]. The lower Moran’s I on T_1_W images (Table 4) represents a spatial redistribution of tissue components with distinct T_1_ relaxation properties, such as viable tumour, proteinaceous regions arising from haemorrhage or tissue degradation and connective tissue replacement. T_2_ signal hyperintensity (Figure 4b) can be attributed primarily to increased free water content with a longer spin–spin relaxation time, for example, under conditions of peritumoural oedema and tumour necrosis [92]. A possible explanation for the marked T_2_W homogeneity (Table 4) is the development of necrotic regions that appeared relatively uniform at MRI’s millimetre-scale resolution, together with regression of the irregular peritumoural oedema noted in the control group. The lower T_2_W Moran’s I values in the FDOX + IMH and LDOX + IMH groups than in the corresponding drug-alone groups imply that IMH partly attenuated the drug-associated increase in T_2_W spatial homogeneity, perhaps through changes in the intratumoural distribution of free water.

At the cellular level, the greatest decline in Moran’s I in H&E-stained images (Figure 5, Table 5 and Table 6) was found after FDOX + IMH, corresponding to a spatially disordered distribution of the dyes within residual viable and non-viable tumour cells and connective tissue fibres. Interestingly, the addition of IMH to FDOX and LDOX produced different heterogeneity patterns. Moran’s I was higher in the LDOX + IMH than in the LDOX group, indicating a more spatially ordered histological pattern with connective tissue replacement, necrosis and additional fat inclusions or inflammatory changes. A greater extent of fibrosis after neoadjuvant therapy has generally been linked to improved survival in patients with soft-tissue sarcoma [79]. When considering the prognostic relevance of necrosis, it is necessary to distinguish spontaneous necrosis as a result of tumour hypoxia from treatment-induced necrosis. The former is characteristic of higher sarcoma grade and, consequently, a poorer prognosis, whereas the latter often has favourable therapeutic implications [93]. In principle, image heterogeneity analysis of histological sections could provide additional quantitative information to differentiate these forms of necrosis based on their spatial heterogeneity patterns.

The therapeutic performance of nanomedicines such as liposomes and polymeric and magnetic nanoparticles is strongly influenced by spatial and temporal heterogeneity within the tumour microenvironment. In sarcomas, inadequate or uneven perfusion leads to limited drug delivery to the tumour core. Variable vascular permeability can alter nanoparticle extravasation from the systemic circulation. A dense extracellular matrix and heterogeneous stromal architecture may further impede penetration through tumour tissue. Elevated interstitial fluid pressure is also known to reduce local drug transport. Taken together, these factors result in non-uniform intratumoural accumulation of LDOX and exposure of sarcoma cells to the drug [94,95,96,97]. They also raise the question of whether treatment-induced spatial organisation of the tumour at the cellular level could depend on formulation-specific mechanisms of drug delivery and distribution. FDOX delivery is mainly mediated by diffusion across the cell membrane. By comparison, LDOX uptake requires either direct fusion of liposomes with the plasma membrane, followed by release of the drug into the cytoplasm, or endocytosis and subsequent intracellular degradation of the liposome. The more complex drug delivery pathway of LDOX limits its immediate antitumour effect on sarcoma cells yet promotes greater retention in the tumour interstitium [98,99]. In fact, the relatively large size of the nanoparticles (~100 nm) restricts their penetration to within one or two tumour cell layers from blood vessels [100]. It is possible that IMH improved LDOX delivery by enhancing the permeability of liposomal and cellular phospholipid bilayers through altered ion transport, reactive oxygen species generation and electromechanical deformation [101,102,103,104]. At the same time, IMH could facilitate FDOX and LDOX delivery by increasing local perfusion and vascular permeability within the tumour region [105]. However, we cannot exclude the possibility that the protonated (cationic) form of FDOX at physiological pH is also influenced by nonthermal effects of the applied electromagnetic field, potentially promoting its electrostatic interactions with the cell membrane [106].

Despite the focus of the current study on changes in intratumoural heterogeneity rather than therapeutic efficacy, one explanation for the relatively strong effect of IMH alone and combined with FDOX or LDOX (Figure 2, Table 3) is that treatment was started during the early avascular phase of sarcoma development (0.2–2 mm in diameter) [107,108]. In the absence of functional tumour vasculature, limited and spatially heterogeneous delivery of systemically administered chemotherapeutic drugs to spindle-like sarcoma-45 cells might have contributed to the comparatively weaker antitumour effect of FDOX or LDOX alone. Nevertheless, the applied electromagnetic field does not rely on vascular transport for tissue penetration and may therefore exert biological effects from the onset of treatment. This interpretation is consistent with prior evidence that hyperthermia alone exhibited antitumour activity and enhanced the uptake or tumour delivery of LDOX when used in combination treatment [109,110].

At the molecular level, analysis of Ki-67-stained images (Table 6) showed increased Moran’s I in all treatment groups, with the highest value following LDOX + IMH. This group also exhibited the lowest Ki-67 staining intensity (Figure 7a). Ki-67 is a marker of cell proliferation. During mitosis, it forms a positively charged coating on chromosome surfaces, preventing the aggregation of chromosome arms through electrostatic repulsion and assisting the symmetrical distribution of nucleolar components between daughter cells [111]. Alterations in the distribution of proliferating cells and associated tissue organisation are likely to underlie the changes in the spatial pattern of Ki-67 staining.

In p53-stained images, the most pronounced increase in Moran’s I occurred after FDOX + IMH treatment (Table 6), whereas the lowest level of p53 was recorded in the LDOX + IMH group (Figure 7b). Though FDOX and LDOX alone had similar effects on the spatial distribution of p53, their combination with IMH resulted in substantially different staining patterns (*p* < 0.05), potentially reflecting differences in tumour cell stress responses and tissue remodelling. An earlier study reported that a more diffuse p53 distribution on immunohistochemical images was linked to poorer prognosis in patients with soft-tissue sarcoma [112]. p53 regulates cell-cycle arrest, DNA repair and apoptosis in response to cellular stress. Disruptions in p53-mediated signalling pathways have been implicated in tumour progression and sensitivity to chemotherapy [113]. Both IMH and doxorubicin act on p53-mediated stress responses through heat and redox mechanisms [114,115]. High expression of Ki-67 and p53 has previously been identified as an adverse prognostic feature in patients with soft-tissue sarcoma [116].

**Figure 9:**
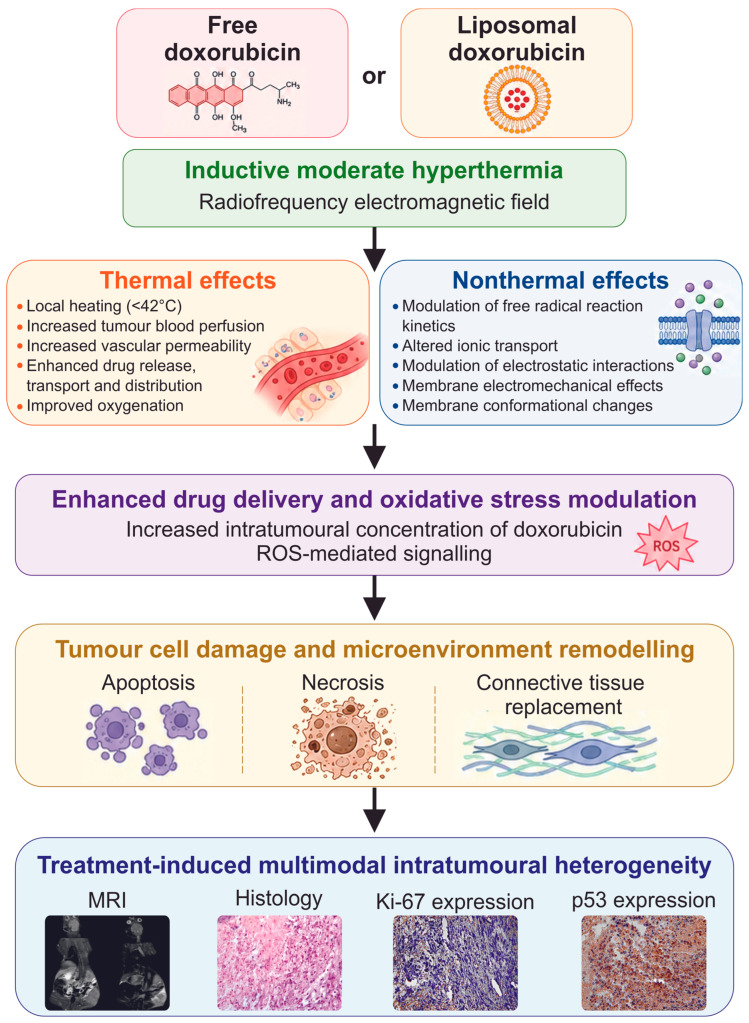
Proposed mechanism underlying treatment-induced changes in multimodal intratumoural heterogeneity of sarcoma-45 in response to FDOX and LDOX combination with IMH.

Figure 9 schematically summarises mechanisms reported in previous experimental and preclinical studies that may give rise to treatment-associated changes in intratumoural sarcoma-45 heterogeneity. A moderate hyperthermia regimen (<42 °C) is well-known to increase tumour blood flow, oxygen and drug delivery. In addition, moderate hyperthermia has been reported to promote local release of doxorubicin from its liposomal carrier [117]. Alongside thermal effects, radiofrequency electromagnetic fields have been shown to influence ionic transport and free radical reaction kinetics, thereby enhancing the reactive oxygen species-mediated signalling and oxidative stress damage caused by doxorubicin [37,118]. Consequently, treatment-induced changes in drug distribution, oxidative stress, tumour destruction and connective tissue replacement can be evident as altered texture heterogeneity on MRI and histological and immunohistochemical images. Our findings are consistent with reports that inductive hyperthermia produces deeper and more uniform heating than convection-based methods while also initiating nonthermal effects associated with electromagnetic fields [119].

Although tumour size remains a widely used criterion for evaluating treatment response, it provides only a macroscopic measure of tumour burden and may not capture sarcoma remodelling, in which necrosis and connective tissue replacement can occur without a change in size [90,120]. Multimodal analysis of intratumoural heterogeneity using MRI, histology and immunohistochemistry offers complementary information on treatment-induced spatial reorganisation at tissue, cellular and molecular levels [121]. It is far from clear, however, how stochastic changes initiated by IMH alone, or combined with FDOX or LDOX, converge across these levels to produce a deterministic tumour response. Within the tumour hierarchy, perturbations at a lower level, for example, the molecular level, may exhibit nonlinear or apparently discordant patterns at a higher level, such as the cellular level. Conversely, changes at a higher level, such as the tissue level, influence organisation at lower levels. Opposite directions of change in Moran’s I across imaging modalities are not necessarily contradictory: treatment may reduce heterogeneity at one organisational level while increasing it at another [122]. The divergent patterns observed following FDOX, LDOX and their combinations with IMH may therefore represent the complex, nonlinear organisation of treatment-induced sarcoma-45 remodelling [123]. Within this framework, FDOX + IMH and LDOX + IMH could have altered not only the extent of sarcoma tissue damage but also the spatial organisation of residual tumour cells and microenvironment components.

Some limitations need to be acknowledged. First, this study was restricted to a single in vivo tumour model. Sarcomas are a highly heterogeneous group of malignant tumours with substantial variation in genetic stability, epigenetic modification, lineage of differentiation, tissue architecture, vascularisation, metabolism, and chemo- and radiosensitivity [124]. The sarcoma-45 model offers a useful experimental platform for evaluating treatment-associated changes relevant to high-grade undifferentiated pleomorphic sarcoma, one of the most common adult soft-tissue sarcoma subtypes [29,31]. For this reason, molecular assessment employed broadly applicable markers. The inclusion of apoptosis-specific (e.g., cleaved caspase-3), angiogenesis (e.g., CD31 and VEGF), and oxidative stress biomarkers and transcriptomic profiling would provide further insights. These findings should be extrapolated selectively to other sarcoma subtypes. Detailed intratumoural temperature mapping and evaluation of temperature variability between animals were beyond the scope of the present study, given imaging artefacts and the lack of MRI–IMH hybrid systems [125]. To supplement the reported *p*-values, epsilon-squared effect sizes (ε^2^) were calculated for multiple-group comparisons. Effect-size measurement helps to characterise the magnitude and potential biological relevance of treatment-associated differences [126]. In the present study, most analysed endpoints showed moderate-to-large effect sizes (0.12–0.59) [127], supporting the consistency of the observed multimodal changes.

Second, image texture analysis was restricted to Moran’s spatial autocorrelation index. Moran’s I compares intensity values at neighbouring locations, relative to the mean intensity of the tumour ROI and is thus applicable for assessing whether imaging phenotypes are spatially clustered or dispersed [74,128]. Alternatively, grey-level co-occurrence matrix (GLCM) descriptors are obtained by counting the frequency with which grey-level pairs occur at a predefined distance and orientation [129]. They summarise local pairwise texture relationships rather than directly measuring global spatial autocorrelation within a non-uniform tumour ROI. The biological relevance of spatial autocorrelation metrics [130] is supported by earlier studies showing that Moran’s I can quantify intratumoural heterogeneity, as reflected in MRI and PET phenotypes at the macroscale [73,75,131,132], as well as in tumour microenvironment composition and gene expression at the microscale [133,134,135,136,137]. Changes in Moran’s I have been previously associated with treatment response or patient prognosis in several cancer types [71,82,137,138,139]. Complementary scale-sensitive and topological analyses, including fractal dimension, lacunarity, coherency and orientation, could additionally characterise treatment-induced structural variation across different levels of tumour organisation. When combined with common prognostic factors, relevant texture parameters allow for better prediction of early treatment response [73]. Yet, further translation of these findings to human sarcomas has to address a number of challenges because imaging-derived tumour heterogeneity biomarkers require standardised acquisition protocols, reproducible segmentation, robust post-processing methods and validation [72]. There is, therefore, a definite need for a multimodal approach to establish clinically meaningful outcomes for data acquired at different levels of tumour organisation, bringing together sarcoma biologists, biomedical engineers, pathologists, radiologists and medical oncologists.

As the present work did not primarily focus on long-term survival, future work involving larger numbers of animals should determine whether the observed treatment-induced image heterogeneity patterns are associated with improved prognosis. The obtained findings suggest that combining non-ionising radiofrequency electromagnetic irradiation as a treatment-enhancing modality with MRI-based assessment may help link biophysical tumour modulation to clinically relevant imaging markers of response. Overall, these limitations highlight priorities for future studies aimed at advancing multimodal assessment of treatment-associated sarcoma heterogeneity.

Finally, fundamental questions still surround the interaction between treatment-induced changes in heterogeneity at the tissue, cellular and molecular levels of tumour organisation. Given that a malignant tumour behaves as a nonlinear dynamic system, the resulting treatment effect is not necessarily equivalent to the sum of changes in the directions of intratumoural heterogeneity observed at any one scale. The principal challenge is to determine quantitatively how these levels are interrelated in shaping the overall sarcoma response to treatment. Addressing this challenge will be essential to developing more informative diagnostic approaches and more effective spatially targeted therapeutic strategies for sarcomas.

## 5. Conclusions

This study showed that the combination of IMH with either FDOX or LDOX induced distinct patterns of intratumoural heterogeneity in sarcoma-45, as assessed by MRI, histological and immunohistochemical image analyses. Divergent changes in Moran’s spatial autocorrelation index in T_1_W and T_2_W MRI, and H&E-, Ki-67- and p53-stained images indicated that the two combination treatments affected the spatial organisation of residual tumour tissue differently at the tissue, cellular and molecular levels. FDOX + IMH produced the greatest overall deviation in multimodal spatial organisation relative to the untreated control group, whereas LDOX + IMH was associated with the lowest tumour growth factor and distinct microscopic patterns characterised by connective tissue replacement and low levels of Ki-67 and p53 staining. However, the inhibitory effect of LDOX + IMH on sarcoma-45 growth kinetics did not differ significantly from that of IMH alone. Collectively, these findings support the view that treatment response cannot be fully characterised by tumour size assessment alone. As this approach provides additional quantitative information from radiology and pathology imaging modalities commonly used in clinical oncology, it may contribute to a more comprehensive evaluation of treatment responses based on intratumoural heterogeneity.

## Figures and Tables

**Table 1:** Maximum specific absorption rate and temperature values during IMH.

Parameter	Skin	Subcutaneous Tissue	Tumour
Specific absorption rate, W/kg	4.64	3.15	4.74
Temperature, °C	39.31	39.26	39.04

**Table 2:** MRI acquisition parameters.

Parameter	T_1_W Image	T_2_W Image
Field strength (T)	1.5	
Imaging frequency (MHz)	63.9	
Repetition time (ms)	500	4035
Echo time (ms)	18	100
Matrix (pixels)	476 × 321	256 × 199
Number of signal averages	1	2
Slice thickness (mm)	2	2
Flip angle (°)	90	
Specific absorption rate (W/kg)	2.5	2.9

**Table 3:** Treatment effect on sarcoma-45 growth kinetics 19 days after tumour inoculation (M ± m).

No.	Group	Growth Factor φ, day^−1^	Breaking Ratio κ, r.u.
1	Control (no treatment)	0.58 ± 0.02	1.00
2	IMH	0.39 ± 0.01 ^a^	1.47
3	FDOX	0.46 ± 0.01 ^ab^	1.24
4	FDOX + IMH	0.43 ± 0.01 ^abc^	1.35
5	LDOX	0.48 ± 0.01 ^abcd^	1.20
6	LDOX + IMH	0.38 ± 0.01 ^acde^	1.52

^a^ Statistically significant difference from control (no treatment), *p* < 0.05; ^b^ statistically significant difference from IMH, *p* < 0.05; ^c^ statistically significant difference from FDOX, *p* < 0.05; ^d^ statistically significant difference from FDOX + IMH, *p* < 0.05; ^e^ statistically significant difference from LDOX, *p* < 0.05.

**Table 4:** Moran’s spatial autocorrelation index in sarcoma-45 tumour and contralateral muscle ROIs on T_1_W and T_2_W MRI scans (M ± m).

No.	Group	Moran’s I T_1_W Image, a.u.	Moran’s I T_2_W Image, a.u.
Tumour ROI			
1	Control (no treatment)	0.76 ± 0.01 *	0.57 ± 0.01 *
2	IMH	0.52 ± 0.01 *^a^	0.42 ± 0.01 *^a^
3	FDOX	0.64 ± 0.01 *^ab^	0.73 ± 0.01 *^ab^
4	FDOX + IMH	0.61 ± 0.01 *^abc^	0.66 ± 0.01 *^abc^
5	LDOX	0.67 ± 0.01 *^abd^	0.72 ± 0.01 *^abd^
6	LDOX + IMH	0.56 ± 0.01 *^acde^	0.65 ± 0.01 *^abce^
		**Muscle ROI**	
	All groups	0.34 ± 0.01	

* statistically significant difference from the muscle ROI in the corresponding group, *p* < 0.05; ^a^ statistically significant difference from control (no treatment), *p* < 0.05; ^b^ statistically significant difference from IMH, *p* < 0.05; ^c^ statistically significant difference from FDOX, *p* < 0.05; ^d^ statistically significant difference from FDOX + IMH, *p* < 0.05; ^e^ statistically significant difference from LDOX, *p* < 0.05.

**Table 5:** Histopathological features of sarcoma-45 damage and tissue replacement.

Feature	Control	IMH	FDOX	FDOX + IMH	LDOX	LDOX + IMH
Necrosis	2	3	1	0	3	2
Apoptosis	2	0	1	2	3	1
Connective tissue replacement	0	3	2	3	1	2
Fatty tissue replacement	0	0	0	0	0	1
Inflammation	0	0	0	0	0	1
Total	4	6	4	5	7	7

Scores: 0—not observed; 1—mild; 2—moderate; 3—severe.

**Table 6:** Moran’s spatial autocorrelation index in sarcoma-45 histological and immunohistochemical images (M ± m).

No.	Group	Moran’s I, a.u.		
		H&E Images	Ki-67 Images	p53 Images
1	Control (no treatment)	0.18 ± 0.01	0.25 ± 0.01	0.33 ± 0.01
2	IMH	0.38 ± 0.01 ^a^	0.32 ± 0.01 ^a^	0.35 ± 0.01
3	FDOX	0.21 ± 0.01 ^b^	0.42 ± 0.01 ^ab^	0.40 ± 0.01 ^ab^
4	FDOX + IMH	0.11 ± 0.01 ^abc^	0.39 ± 0.01 ^abc^	0.68 ± 0.01 ^abc^
5	LDOX	0.25 ± 0.01 ^abcd^	0.38 ± 0.01 ^abcd^	0.40 ± 0.01 ^abd^
6	LDOX + IMH	0.30 ± 0.01 ^abcde^	0.48 ± 0.01 ^abcde^	0.38 ± 0.01 ^abcd^

^a^ Statistically significant difference from control, *p* < 0.05; ^b^ statistically significant difference from IMH, *p* < 0.05; ^c^ statistically significant difference from FDOX, *p* < 0.05; ^d^ statistically significant difference from FDOX + IMH, *p* < 0.05; ^e^ statistically significant difference from LDOX, *p* < 0.05.

## Data Availability

The data presented in this study are available upon request from the corresponding author.

## Abbreviations

The following abbreviations are used in this manuscript:

a.u.	Arbitrary units
DNA	Deoxyribonucleic acid
FDOX	Free doxorubicin
H&E	Haematoxylin–eosin–orange
IMH	Inductive moderate hyperthermia
LDOX	Liposomal doxorubicin
MRI	Magnetic resonance imaging
ROI	Region of interest
r.u.	Relative units
SAR	Specific absorption rate
T_1_W	T_1_-weighted
T_2_W	T_2_-weighted
